# Lung adenocarcinoma: selection of surgical approaches in solid adenocarcinoma from the viewpoint of clinicopathologic features and tumor microenvironmental heterogeneity

**DOI:** 10.3389/fonc.2024.1326626

**Published:** 2024-03-05

**Authors:** Xiao Li, Zhen Gao, Haixiao Diao, Chenran Guo, Yue Yu, Shang Liu, Zhen Feng, Zhongmin Peng

**Affiliations:** ^1^ Department of Thoracic Surgery, Shandong Provincial Hospital, Shandong University, Jinan, Shandong, China; ^2^ Department of Thoracic Surgery, Shandong Provincial Hospital Affiliated to Shandong First Medical University, Shandong First Medical University, Jinan, Shandong, China

**Keywords:** lung adenocarcinoma, histological subtype, solid adenocarcinoma, clinicopathological feature, tumor microenvironmental heterogeneity

## Abstract

**Introduction:**

Solid adenocarcinoma represents a notably aggressive subtype of lung adenocarcinoma. Amidst the prevailing inclination towards conservative surgical interventions for diminutive lung cancer lesions, the critical evaluation of this subtype’s malignancy and heterogeneity stands as imperative for the formulation of surgical approaches and the prognostication of long-term patient survival.

**Methods:**

A retrospective dataset, encompassing 2406 instances of non-solid adenocarcinoma (comprising lepidic, acinar, and papillary adenocarcinoma) and 326 instances of solid adenocarcinoma, was analyzed to ascertain the risk factors concomitant with diverse histological variants of lung adenocarcinoma. Concurrently, RNA-sequencing data delineating explicit pathological subtypes were extracted from 261 cases in the TCGA database and 188 cases in the OncoSG database. This data served to illuminate the heterogeneity across lung adenocarcinoma (LUAD) specimens characterized by differential histological features.

**Results:**

Solid adenocarcinoma is associated with an elevated incidence of pleural invasion, microscopic vessel invasion, and lymph node metastasis, relative to other subtypes of lung adenocarcinoma. Furthermore, the tumor microenvironment (TME) in solid pattern adenocarcinoma displayed suboptimal oxygenation and acidic conditions, concomitant with augmented tumor cell proliferation and invasion capacities. Energy and metabolic activities were significantly upregulated in tumor cells of the solid pattern subtype. This subtype manifested robust immune tolerance and capabilities for immune evasion.

**Conclusion:**

This present investigation identifies multiple potential metrics for evaluating the invasive propensity, metastatic likelihood, and immune resistance of solid pattern adenocarcinoma. These insights may prove instrumental in devising surgical interventions that are tailored to patients diagnosed with disparate histological subtypes of LUAD, thereby offering valuable directional guidance.

## 1 Introduction

Lung cancer remains the preeminent cause of cancer-related mortality and ranks as the second most frequently diagnosed cancer globally (1). Among its histopathologic variants, lung adenocarcinoma (LUAD) is predominant (2). The International Multidisciplinary Lung Adenocarcinoma Classification, a collaborative initiative by the International Association for the Study of Lung Cancer, American Thoracic Society, and European Respiratory Society (IASLC/ATS/ERS), categorizes LUAD into five histological subtypes: lepidic, acinar, papillary, micropapillary, and solid adenocarcinoma (3), the 2015 and 2021 WHO Classification of Lung Tumors also supports this viewpoint (4, 5). Accumulated evidence underscores considerable heterogeneity among these LUAD subtypes, with solid pattern adenocarcinoma notably linked to poor prognostic outcomes (6–9).

Surgical resection stands as the standard of care for early-stage lung cancer, with pulmonary lobectomy being the conventional surgical procedure (10). Advances in high-resolution computed tomography (CT) scans have augmented the detection rates of small-sized lung cancers, thereby influencing evolving surgical management paradigms (11–13). According to the CALGB140503 and JCOG0802 studies, sub-lobectomy may suffice as an effective, if not standard, surgical procedure for small-sized peripheral lung cancers (14, 15). Similarly, the JCOG1211 study established the safety and efficacy of lung segmental resection for patients with ground-glass opacity (GGO) non-small cell lung cancer (NSCLC) having a tumor diameter of 3 cm or less, even when GGO exceeds 2 cm (16). However, these landmark studies have not accounted for the heterogeneity intrinsic to various LUAD pathological subtypes. Hence, devising surgical approaches based solely on nodule dimensions may not universally benefit patients. Specifically, pulmonary sub-lobectomy, as opposed to lobectomy, may elevate the risk of tumor recurrence in cases of highly malignant micropapillary and solid adenocarcinomas. Substantiating this are studies indicating that a solid pattern component serves as an independent prognostic marker for early recurrence in stage I LUAD, even in NSCLCs that are 2 cm or smaller in diameter (17, 18). The suitability of sub-lobectomy for LUADs with diameters ≤2cm and solid components, therefore, remains a topic necessitating further inquiry.

Given that prior research has chiefly addressed the clinicopathological features and molecular underpinnings of larger LUAD lesions (19–21), a nuanced analysis of the tumor attributes and tumor microenvironment (TME) among LUADs with diameters ≤2cm could enrich our understanding of their invasive and metastatic behaviors. Such insights could subsequently inform the formulation of surgical plans tailored for small-sized lung cancers, aiming to minimize recurrence and enhance patient survival rates. In this study, we scrutinized risk factors associated with pleural invasion, spread through air spaces (STAS), microscopic vessel invasion, and lymph node metastasis in LUADs ≤2cm. Furthermore, we explored the heterogeneity of the TME across varying pathological subtypes via transcriptomic analysis of LUAD samples. Our analyses of transcriptomic data reveal new facets of the aggressive nature, immune evasion capabilities, and metabolic preferences of solid pattern adenocarcinoma. These findings have been corroborated through multi-cohort transcriptomic data sets, offering a compelling foundation for the development of surgical strategies based on histological subtypes for early-stage, small-sized lung cancers.

## 2 Materials and methods

### 2.1 Patients

This retrospective study involved a comprehensive review and analysis of patients who underwent surgical intervention for primary LUAD at our institution between January 2018 and December 2022. Ethical clearance was secured from the institutional review board, and informed consent was duly obtained from each patient prior to surgical procedures. Patient inclusion and exclusion criteria are comprehensively detailed in **Supplementary Table S1**. Transcriptomic data pertinent to LUAD were procured from the TCGA database (https://portal.gdc.cancer.gov/) and the OncoSG database (https://src.gisapps.org/OncoSG/). The criteria for identifying solid pattern adenocarcinoma samples were delineated by the study conducted by Zhong-Yi Dong et al (20). Patient inclusion and exclusion criteria are comprehensively detailed in **Supplementary Tables S2**, **S3**. All transcriptomic data were converted to transcripts per million reads (TPM) format and subsequently underwent log2 transformation.

### 2.2 Histological assessment

Surgical specimens were independently evaluated by two pathologists, one with over a decade of experience and another with more than five years. Both were blinded to clinical data. In instances of discordant evaluations, a third pathologist was consulted to reach a consensus. Tumor classification was conducted in adherence to the most recent definitions stipulated by the World Health Organization (WHO) (4). Each LUAD pathological subtype was quantified in a semi-quantitative manner, incremented at 5% levels, cumulatively totaling 100% subtype components per tumor (3, 4). High-risk solid subtypes were defined as those containing at least 5% solid components (22, 23). In cases of mixed-type LUAD, the most prevalent pattern was identified as the subtype constituting the majority of the tumor, with a minimum threshold set at 30% (3).

### 2.3 Identification and enrichment analysis of differentially expressed genes

Differential gene expression analyses were conducted utilizing the limma package. An adjusted *P*-value <0.05 and a log2|fold change (FC)| >1 were established as criteria for identifying significantly differentially expressed genes (DEGs). Subsequent enrichment analyses, comprising Gene Ontology (GO) and Kyoto Encyclopedia of Genes and Genomes (KEGG) evaluations, were facilitated via the ClusterProfiler package. The specified parameters included minGSSize=10 and species=Homo sapiens. In the Gene Set Enrichment Analysis (GSEA), results were deemed significant if the false discovery rate (FDR) was <0.25, the *P*-value <0.05, and the normalized enrichment score (|NES|) >1.

### 2.4 Differential expression analysis and gene set variation analysis

The GSVA package in R (version 1.32.0) was employed to calculate gene set enrichment scores across sample groups (24). Between-group comparisons of gene set enrichment scores were performed using the Wilcoxon rank-sum test. Differential activity of gene sets across varied groups was computed through the limma package. Benjamini-Hochberg correction was applied to adjust *P*-values, which were set at <0.05 for identifying significantly altered gene sets. Source databases for KEGG gene sets, Hallmark gene sets, C5 gene sets, and Metabolic process gene sets included the KEGG GENES Database (https://www.genome.jp/kegg/genes.html). Detailed gene lists and references for parameters such as Tumor proliferation rate, Hypoxia, Glycolysis, Lactate transmembrane transporter activity, Checkpoint molecules, Ubiquitin mediated proteolysis, One carbon pool by folate, Galactose metabolism, Macrophage and dendritic cell traffic, Fibrillar collagens, Matrix remodeling, Epithelial-mesenchymal transition (EMT), and EMT signature gene sets are available in **Supplementary Table S6**. The tumor stemness score was determined via the Tathiane M scoring system, as applied to TCGA samples (25).

### 2.5 Tumor microenvironmental immune scoring and ssGSEA immune cell annotation

The Estimate package was employed to quantify immune cell infiltration levels within the tumor microenvironment (https://bioinformatics.mdanderson.org/estimate/index.html). Single-sample gene set enrichment analysis (ssGSEA) is principally invoked when conventional GSEA is unsuitable for individual samples. This algorithm comprises two key steps: initial rank-normalization of gene expression values for the sample in question, followed by the computation of the enrichment score via the empirical cumulative distribution function (26). Utilizing an immune cell signature gene set proposed by Charoentong, P. et al. (27, 28), the GSVA package facilitated the quantification of 28 distinct types of immune cell infiltrates in the tumor microenvironment. The relative levels of infiltration for each immune cell type were characterized by ssGSEA-derived enrichment scores, normalized to a uniform distribution spanning from 0 to 1.

### 2.6 Survival analysis

Single-factor Cox regression survival analysis was executed for each variable independently. Kaplan-Meier survival curves were employed to visualize disparities in survival rates across distinct groups. Significance testing for survival rates between groups was conducted using the log-rank test. The analyses were facilitated using the “survival” and “forestplot” packages in R. Results were summarized and visualized via the “survminer” R package.

### 2.7 Statistics analysis

Statistical evaluations were performed using R software, version 4.2.2. For quantitative data adhering to a normal distribution, a t-test was applied. Non-normally distributed data were analyzed using the Wilcoxon test. In scenarios involving multi-group analysis, the Kruskal-Wallis test was employed for nonparametric evaluations, whereas analysis of variance (ANOVA) was utilized for parametric assessments (29). Event rates were ascertained via Fisher’s exact test. Prognostic differences between groups were analyzed using the “survival” R package, with the log-rank test applied to determine the statistical significance of differing prognoses among disparate groups. A two-sided *P*-value of less than 0.05 was deemed statistically significant. The Benjamini-Hochberg method was employed for controlling the false discovery rate (FDR) during multiple hypothesis testing (30).

## 3 Results

### 3.1 Demographic and clinicopathological features

**Table 1** delineates the demographic and clinicopathological attributes of the study’s final cohort. In the non-solid pattern group, the median age stood at 58 years, juxtaposed against a median age of 59.5 years in the solid pattern group. This age variation between the two groups did not reach statistical significance (*P*=0.08). Contrastingly, notable differences were manifested in gender distribution, featuring a male predominance in the solid pattern group (57.06% vs. 37.41%, *P*<0.001). Smoking status further discriminated between the groups, with a significantly greater proportion of smokers found in the solid pattern group (42.63% vs. 22.73%, *P*<0.001). When evaluated for P-d levels, the solid pattern group displayed a higher median value (1.60 vs. 1.50, *P*<0.001). Moreover, significant variances were evident between the two groups with respect to pleural invasion (15.03% vs. 1.5%, *P*<0.001), STAS (7.06% vs. 1.04%, *P*<0.001), microscopic vessel invasion (4.91% vs. 0.02%, *P*<0.001), and lymph node metastasis (N1: 11.96% vs. 1.00%; N2: 18.71% vs. 1.91%, *P*<0.001). Clinicopathological features of both the TCGA and OncoSG cohorts are concisely presented in **Supplementary Tables S4**, **S5**, respectively. In summary, these data substantiate that lung adenocarcinoma (LUAD) is more prevalent among females, whereas its solid variant is disproportionately represented among smoking males. Additionally, the solid pattern is characterized by a greater maximum pathology diameter and higher incidences of pleural invasion, microscopic vessel invasion, STAS, and lymph node metastasis, all of which were statistically significant.

**Table 1:** Validation-LUAD Cohort demographic and clinicopathological characteristics of patients.

Characteristics	Other types(N=2406)	Solid (N=326)	Total(N=2732)	*P* value
**Age**				0.08
Median[min-max]	58 [23, 82]	60 [26, 79]	58 [23, 82]	
**Gender**				<0.001
Female	1506(62.59%)	140(42.94%)	1646(60.25%)	
Male	900(37.41%)	186(57.06%)	1086(39.75%)	
**Smoking**				<0.001
Never	1859(77.27%)	187(57.36%)	2046(74.89%)	
Current/Ever	547(22.73%)	139(42.63%)	686(25.11%)	
**P-d**				<0.001
Median[min-max]	1.50[0.50,2.00]	1.60[0.50,2.00]	1.50[0.50,2.00]	
**Pleural invasion**				<0.001
Absent	2370(98.50%)	277(84.97%)	2647(96.89%)	
Present	36(1.50%)	49(15.03%)	85(3.11%)	
**STAS**				<0.001
Absent	2381(98.96%)	303(92.94%)	2684(98.24%)	
Present	25(1.04%)	23(7.06%)	48(1.76%)	
**Microscopic vessel invasion**				<0.001
Absent	2401(99.72%)	310(95.09%)	2711(99.23%)	
Present	5(0.02%)	16(4.91%)	21(0.77%)	
**Lymphatic metastasis**				<0.001
N0	2336(97.09%)	226(69.33%)	2562(93.78%)	
N1	24(1.00%)	39(11.96%)	63(2.31%)	
N2	46(1.91%)	61(18.71%)	107(3.92%)	

Data are numbers of patients, with percentages in parentheses. Other = Other pattern adenocarcinomas (lepidic adenocarcinomas (n=398), acinar adenocarcinomas (n=1770) and, papillary adenocarcinomas (n=193) Invasive mucinous (n=40)); Solid, Solid pattern adenocarcinomas; P-d, Maximum Pathological diameter of tumor; STAS, spread through air spaces.

### 3.2 Significant differences in the tumor microenvironment between solid and non-solid pattern adenocarcinomas

**Figure 1:**
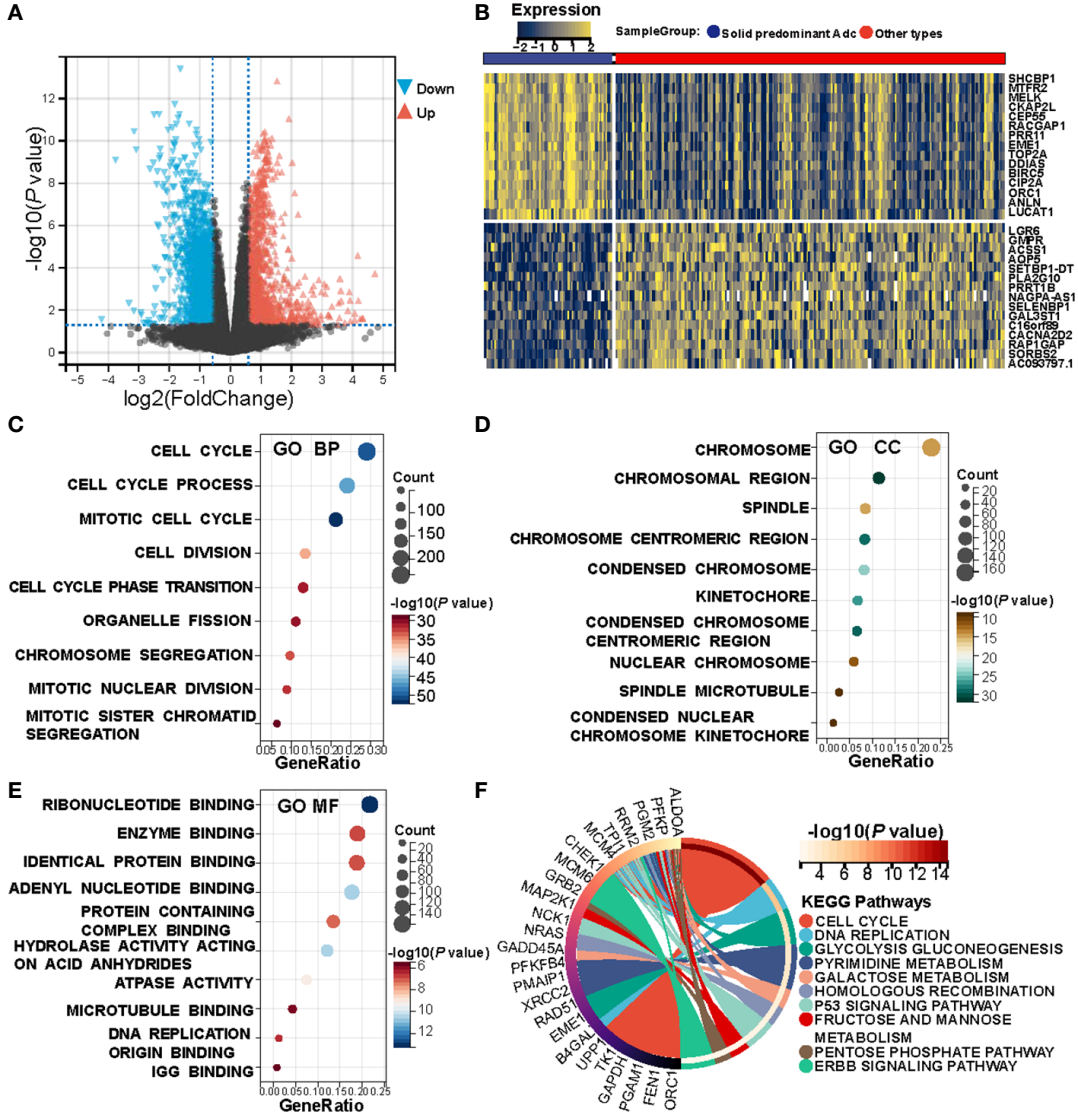
Heterogeneity between solid pattern and other patterns in the TCGA cohort. **(A)** Volcano Plot of differentially expressed genes between the two groups. **(B)** Heatmap of differentially expressed genes between the two groups. **(C–E)** Bubble Charts of biological processes, cellular components, and molecular function analysis of differentially expressed genes, with the size of each point representing the number of enriched genes, and the shade of color indicating the level of significance. **(F)** KEGG Pathway Enrichment Chord Diagram shows that the solid pattern is mainly associated with active cellular process, nucleotide metabolism, and more aggressive biomarkers.

The prior analysis substantiates that solid pattern adenocarcinoma displays a more aggressive clinicopathological profile. To delve further into this observation, we queried the TCGA database, acquiring 62 instances of solid pattern adenocarcinoma and 199 of other LUAD subtypes for a comprehensive differential gene expression analysis. Distinct disparities between the solid pattern and other LUAD subtypes were delineated by the volcano plot (**Figure 1A**) and the heat map (**Figure 1B**). Specifically, a total of 883 genes were found to be highly expressed, while 517 genes displayed low expression levels in the solid pattern adenocarcinoma; further details are catalogued in **Supplementary Table S6**. Subsequent functional enrichment analysis focused on the highly expressed genes revealed several associations. Biological Process (BP) analysis indicated that the solid pattern adenocarcinoma is predominantly characterized by an activated cell cycle, corroborated by **Figure 1C**. Cellular Component (CC) analysis underscored that chromosomes and mitotic processes are central features, as substantiated by **Figure 1D**. Furthermore, Molecular Function (MF) analysis revealed a primary association with nucleotide metabolism, as illustrated in **Figure 1E**. High-throughput KEGG pathway analysis highlighted that the highly expressed genes are principally involved in active cellular mechanisms, nucleotide metabolism, and invasive markers (Pathways: Cell Cycle, Pyrimidine Metabolism, Galactose Metabolism, p53 Signaling Pathway, ERBB Signaling Pathway), as demonstrated in **Figure 1F**.

### 3.3 Solid pattern adenocarcinoma exhibits enhanced cell proliferation and invasiveness

**Figure 2:**
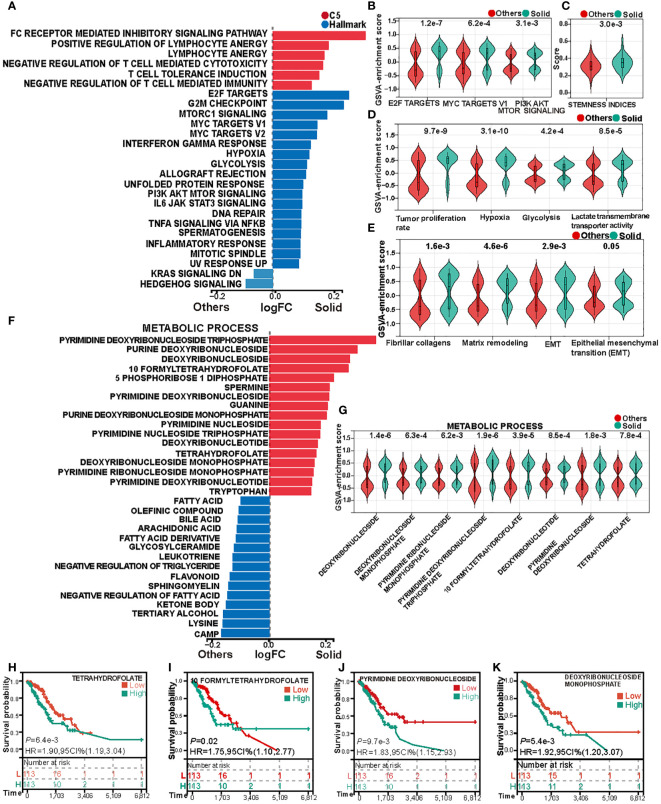
Heterogeneity in cell proliferation, invasive capacity, and metabolism between solid pattern and other patterns in the TCGA cohort. **(A)** Gene Set Variation Analysis (GSVA) reveals differences in the enrichment scores of Hallmark gene sets and C5 gene sets between the two groups. **(B)** Violin Plot shows differences in the enrichment scores of biomarkers associated with invasion and metabolism between the two groups. **(C)** Violin Plot shows differences in tumor stemness scores between the two groups. **(D)** Violin Plot shows the differences in cell proliferation rate, hypoxia, glycolysis and lactate transmembrane transporter activity scores between the two groups. **(E)** Violin Plot shows the differences in collagen fiber transcription score and extracellular matrix remodeling score between the two groups. **(F)** Gene Set Variation Analysis (GSVA) reveals significant differences in the enrichment of metabolic process gene sets between the two groups. **(G)** Violin Plot shows differences in nucleotide and tetrahydrofolate metabolism between the two groups. **(H–K)** Kaplan-Meier analysis of tetrahydrofolate, formyltetrahydrofolate, pyrimidine deoxyribonucleoside, and deoxyribonucleoside monophosphate.

GSVA was employed to compute the pathway enrichment scores of both C5 gene sets and Hallmark gene sets across each sample in the TCGA cohort. Differential analysis of these scores revealed marked differences between solid pattern and other patterns, as depicted in **Figure 2A**. Notably, the tumor immune activity in solid pattern adenocarcinoma was found to be suppressed. Concurrently, genes correlated with invasiveness and metabolic activity, such as E2F Targets, MYC Targets V1, and PI3K AKT MTOR Signaling, were upregulated within the tumor cells of the solid pattern samples (**Figure 2B**). A similar trend was observed upon analysis of the OncoSG cohort (**Supplementary Figures S1A, B**). These observations imply a more pronounced invasive capability and immune evasion in solid pattern adenocarcinoma.

Prior studies indicate that poorly differentiated primary tumors are generally associated with increased malignancy, characterized by greater invasive and metastatic potential, which often results in accelerated disease progression and poor prognostic outcomes (31, 32). Poorly differentiated tumors also frequently exhibit elevated tumor stemness scores (25). In alignment with this, our analysis showed that solid pattern adenocarcinomas registered higher tumor stemness scores, as evidenced by **Figure 2C**. This finding substantiates the characterization of solid pattern as a poorly differentiated subtype of LUAD, typically associated with adverse clinical outcomes.

Additional GSVA analyses disclosed that hypoxic conditions and glycolytic activity are more prominent in solid pattern adenocarcinomas (**Figures 2A, D**). Further application of GSVA to quantify tumor proliferation rates and lactate transmembrane transporter activity across samples revealed that solid pattern adenocarcinomas had a significantly elevated tumor proliferation rate and increased lactate transmembrane transporter activity (**Figure 2D**). These findings were corroborated in the OncoSG cohort (**Supplementary Figures S1A, C**), supporting the hypothesis that tumor cells in solid pattern adenocarcinoma are more proliferative and engender a more hypoxic and acidic tumor microenvironment.

Previous investigations have established that collagen generation and fibrosis progression in cancer-associated fibroblasts, along with diminished collagen degradation capacity, are factors that contribute to tumor growth, invasion, and chemoresistance (33, 34). Such processes also result in the extracellular matrix (ECM) becoming a mechanical barrier that inhibits immune cell migration and infiltration into the tumor parenchyma (35). In light of these insights, we evaluated the fibrocollagenous transcriptomic score and ECM remodeling score between solid pattern and other patterns (**Figure 2E**). Solid pattern adenocarcinomas exhibited elevated scores for both fibrocollagenous transcriptomic and ECM remodeling activities. Furthermore, they showed increased levels of epithelial-mesenchymal transition (EMT) within the tumor microenvironment (**Figure 2E**). These trends were confirmed in the OncoSG cohort (**Supplementary Figure S1D**). These findings suggest that malignant cells in solid pattern adenocarcinomas are more inclined to undergo EMT, thereby acquiring a more migratory mesenchymal phenotype. This facilitates distal tumor metastasis and contributes to treatment failure (36).

### 3.4 Metabolic process shows significant heterogeneity between solid and non-solid pattern adenocarcinoma

Gene Set Variation Analysis (GSVA) was utilized to compute the enrichment scores for gene sets associated with metabolic processes in each sample within the TCGA cohort. The differential analysis of these scores disclosed pronounced disparities in the activity of various metabolic pathways between solid pattern adenocarcinomas and other patterns, as delineated in **Figure 2F**. Specifically, solid pattern adenocarcinomas manifested heightened nucleotide metabolism, whereas other patterns were characterized by elevated fatty acid, triglyceride, and ketone body metabolism. Furthermore, enhanced tetrahydrofolate metabolism was evident in solid pattern adenocarcinomas (**Figure 2G**). To explore the prognostic implications, we stratified the samples into high and low activity groups, based on the median score for tetrahydrofolate metabolism. Subsequent survival analysis of these stratified groups revealed a considerably worse prognosis for the cohort with elevated tetrahydrofolate metabolic activity (**Figures 2H, I**). In a parallel manner, an assessment of nucleotide metabolic activity disclosed that the subset with heightened activity in this pathway also experienced poorer prognostic outcomes (**Figures 2J, K**, **Supplementary Figure S2**). These findings were corroborated by the OncoSG cohort (**Supplementary Figures S1E–J**). Collectively, these results suggest that solid pattern adenocarcinomas are characterized by an activated cellular proliferation metabolism. Furthermore, folate-mediated one-carbon metabolism emerges as a pivotal factor influencing the survival and proliferative capabilities of the cancer cells (37).

### 3.5 Tumor microenvironment of solid pattern adenocarcinoma exhibits a significant immunosuppressive state

**Figure 3:**
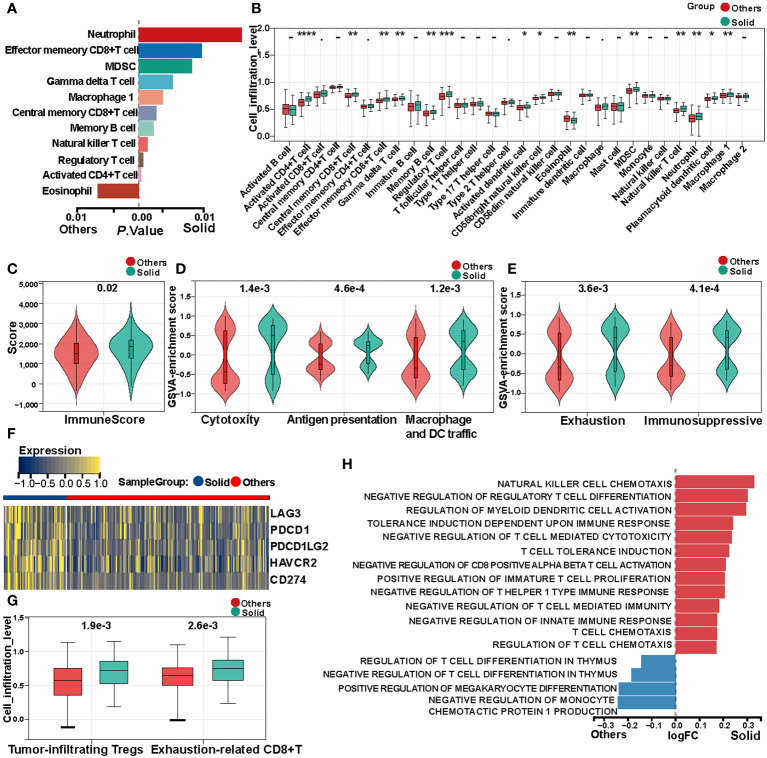
Immune heterogeneity between solid pattern and other patterns in the TCGA cohort. **(A)** Single-sample Gene Set Enrichment Analysis (ssGSEA) reveals differences in immune cell infiltration scores between the two groups. **(B)** Box Plot shows differences in immune cell infiltration abundance between the two groups. **(C)** Violin Plot shows differences in immune scores between the two groups. **(D)** Violin Plot shows differences in cytotoxicity scores between the two groups. **(E)** Violin Plot shows differences in tumor microenvironment immune exhaustion scores between the two groups. **(F)** Heatmap of differentially expressed immune checkpoints between the two groups. **(G)** Box Plot shows differences in quantification of tumor-infiltrating regulatory T cells (Tregs) and exhausted CD8^+^T cell within the tumor microenvironment between the two groups. **(H)** Gene Set Variation Analysis (GSVA) shows significant differences in immune tolerance, immune suppression, and immune cell physiological functions between the two groups. * means "P < 0.05", ** means "P < 0.01", *** means "P < 0.001" , **** means "P < 0.0001".

Single-sample Gene Set Enrichment Analysis (ssGSEA) was utilized to compute the immune cell infiltration scores for each sample within the TCGA cohort. Subsequent differential analysis of these scores disclosed significant disparities in immune cell infiltration among various subtypes (**Figure 3A**, **Supplementary Figure S3A**). Specifically, activated CD4^+^T cells, regulatory T cells (Tregs), natural killer T cells, central memory CD8^+^T cells, and M1 macrophages exhibited elevated infiltration in the solid pattern adenocarcinomas. In contrast, eosinophils demonstrated higher infiltration in other adenocarcinoma patterns (**Figure 3B**, **Supplementary Figure S3B**).

Immune scores for each sample were calculated using the ESTIMATE algorithm. Comparative analysis between solid pattern and other pattern adenocarcinomas indicated that immune scores were significantly elevated in the solid pattern (**Figure 3C**, **Supplementary Figure S3C**). Moreover, cell cytotoxicity, representing the functional efficacy of CD8^+^T cells in tumor eradication, was quantified (38). The data revealed that cytotoxicity levels in the solid pattern were significantly elevated compared to other patterns (**Figure 3D**, **Supplementary Figure S3D**).

Given the pivotal role of antigen-presenting cells in modulating tumor immune responses, we quantified the antigen presentation capacity as well as the infiltration levels of macrophages and dendritic cells. Our findings indicate that the solid pattern is characterized by an enhanced presence of antigen-presenting cells and elevated antigen presentation capabilities.

Despite the aforementioned active immune responses, the solid pattern adenocarcinoma manifests higher recurrence rates and poorer prognostic outcomes compared to other patterns. Accordingly, we calculated both immune exhaustion and immunosuppressive scores for each sample. The analysis corroborated that the solid pattern is in an accentuated state of immune inhibition and exhaustion within the tumor microenvironment (**Figure 3E**, **Supplementary Figure S3E**). Immune checkpoint blockade (ICB) gene analysis further revealed significant upregulation of *LAG3*, *PDCD1*, *PDCD1LG2*, *HAVCR2*, and CD274 in the solid pattern adenocarcinomas (**Figure 3F**, **Supplementary Figure S3F**).

The normal physiological role of Tregs is to maintain immune equilibrium; however, tumor cells can subvert these Tregs to bolster their own growth (39). Quantification of tumor-infiltrating Tregs revealed a significant elevation in the solid pattern compared to other patterns within the tumor microenvironment. Additionally, a higher abundance of exhausted CD8^+^T cells was also observed in the tumor microenvironment of the solid pattern (**Figure 3G**, **Supplementary Figure S3G**).

Lastly, GSVA was applied to compute enrichment scores for immune-related pathways within the C5 gene sets. Differential analysis of these scores substantiated significant differences between the solid pattern and other patterns, with the former primarily displaying features of immune tolerance, immune suppression, and negative regulation of immune cell functions (**Figure 3H**, **Supplementary Figure S3H**).

### 3.6 Solid pattern adenocarcinoma is associated with a poorer prognosis

**Figure 4:**
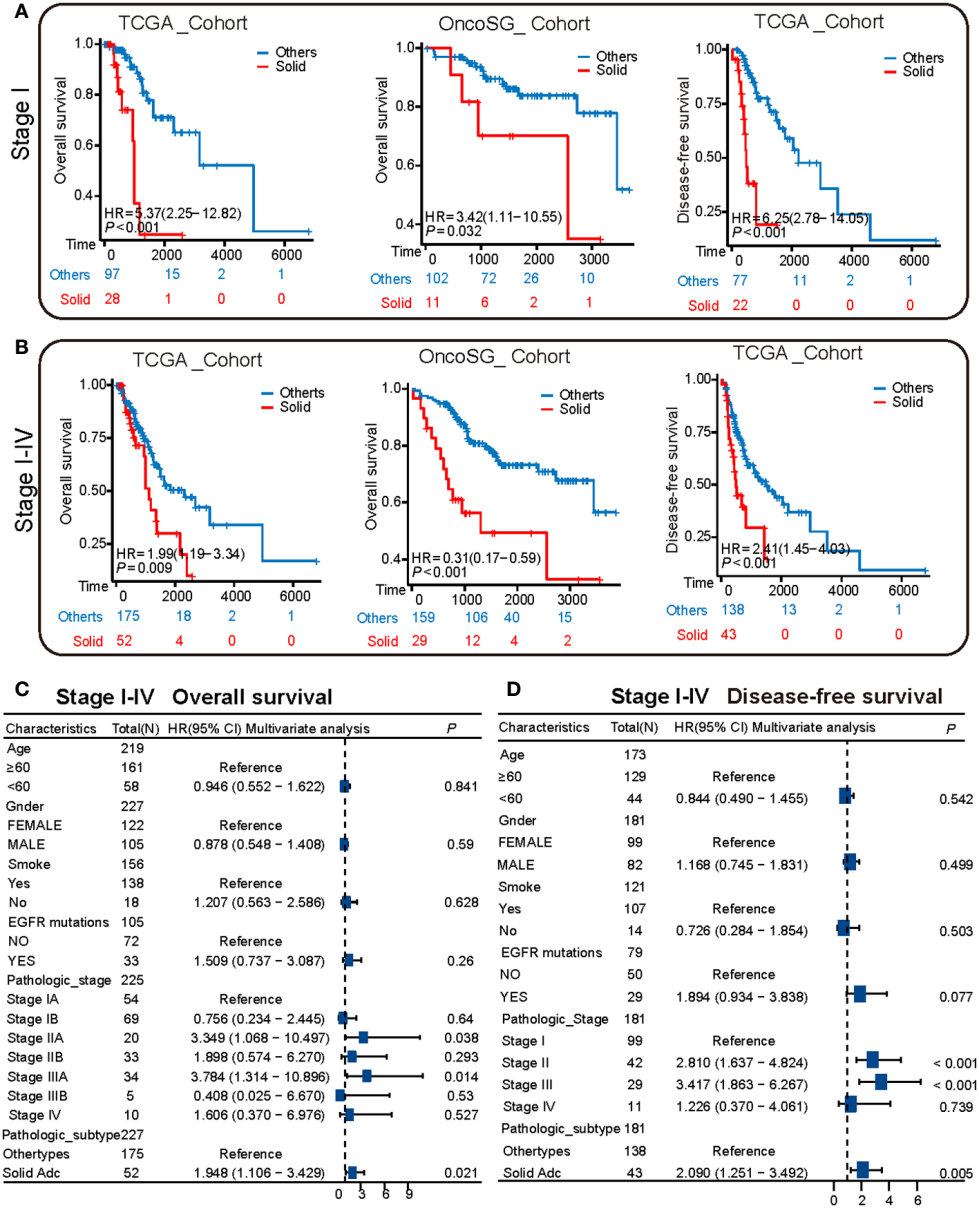
Survival heterogeneity between solid pattern and other patterns in the TCGA cohort. **(A)** The Kaplan-Meier curves depict the overall survival and disease-free survival of stage I patients in both TCGA and OncoSG cohorts. Patients with solid pattern adenocarcinoma have significantly lower overall survival and disease-free survival compared to other patterns. **(B)** The Kaplan-Meier curves depict the overall survival and disease-free survival of stage I to IV patients in both TCGA and OncoSG cohorts. Patients with solid pattern adenocarcinoma have significantly lower overall survival and disease-free survival compared to other patterns. **(C)** Multivariate survival analysis shows that the solid pattern pathological subtype is an independent risk factor for overall survival. **(D)** Multivariate survival analysis shows that the solid pattern pathological subtype is an independent risk factor for disease-free survival.

Kaplan-Meier survival analysis was executed on samples from the TCGA and OncoSG cohorts, specifically focusing on those with pathological stage I. The analytical outcomes indicated that individuals with solid pattern adenocarcinoma manifested a markedly worse overall survival (OS) as well as disease-free survival (DFS) compared to other subtypes (**Figure 4A**). This analysis was extended to encompass patients across pathological stages I to IV. Data corroborated that solid pattern adenocarcinoma is associated with diminished OS and truncated DFS, as illustrated in **Figure 4B**. To further substantiate these findings, multivariable survival analysis was conducted utilizing the Cox proportional hazards regression model. This model revealed that the solid pattern pathological subtype serves as an independent prognostic risk factor for overall survival (**Figure 4C**, **Supplementary Figure S4**). Concurrently, analysis targeting DFS confirmed that the solid pattern pathological subtype persists as an independent risk factor for disease-free survival as well (**Figure 4D**).

## 4 Discussion

The advent of high-resolution computed tomography (CT) has catalyzed the identification of an escalating number of early-stage lung cancers within the population, thereby instigating an evolution in surgical paradigms pertaining to lung cancer treatment (11–13). Specifically, there has been a notable transition from total lung resection or pulmonary lobectomy to more conservative surgical techniques such as limited resections (10, 40, 41). Recent clinical trials, including the CALGB140503 and JCOG0802 studies, postulate that sub-lobectomy constitutes an efficacious, if not standard, surgical procedure for the treatment of diminutive peripheral lung cancers (14, 15). For early-stage non-small cell lung cancer (T1a,b N0 NSCLC), both wedge resection and lung segmental resection are deemed clinically acceptable interventions. However, the surgical decision-making process should not be circumscribed merely by tumor dimensions. It is imperative to consider the inherent risk factors associated with specific pathological subtypes, such as solid pattern adenocarcinoma, which mandates particular vigilance from thoracic surgeons (42). Existing literature, including studies by Nitadori and Su et al., posits that limited resections engender a heightened risk of cancer recurrence relative to pulmonary lobectomy for cases with high-risk pathological subtypes (43, 44). Our own empirical analysis substantiates that small-sized solid pattern adenocarcinoma is significantly associated with elevated incidences of pleural invasion, microscopic vessel invasion, and lymph node metastasis when juxtaposed with other subtypes of lung adenocarcinoma (LUAD). Such adverse oncological events invariably portend a compromised prognosis (45, 46). Further corroborating this point, our survival analyses elucidate that solid pattern adenocarcinoma is a detrimental prognostic factor for early-stage LUAD in terms of both disease-free survival (DFS) and overall survival (OS), thus aligning with extant research findings (6–9). Consequently, an indiscriminate focus on nodule size as the sole criterion for surgical intervention may not necessarily yield optimal outcomes for all patients afflicted with small-sized lung cancer.

To elucidate the underlying molecular dynamics that might guide surgical interventions, we conducted a comprehensive transcriptomic analysis comparing solid pattern adenocarcinoma to other histological subtypes (lepidic, acinar, papillary) in lung adenocarcinoma (LUAD) samples. Our data reveal marked heterogeneity between solid pattern and other pattern adenocarcinomas. Specifically, we discerned an upregulation of genes in the tumor microenvironment of solid pattern adenocarcinoma, predominantly linked to active cellular processes, nucleic acid metabolism, and invasive biomarkers. These results warrant further in-depth scrutiny into the unique characteristics of the tumor microenvironment in solid pattern adenocarcinoma. Notably, poorly differentiated primary tumors generally present with a heightened degree of malignancy, exhibiting increased invasive and metastatic capacities, thereby contributing to disease progression and an adverse prognosis (31, 32). These tumors often manifest elevated tumor stemness scores (25). Additionally, the microenvironment of solid pattern tumors is characterized by distinct biochemical parameters such as acidity and hypoxia. Furthermore, these tumors exhibit enhanced proliferative activity and elevated stemness indices. Beyond this, our data point to an augmented state of nucleic acid metabolism and folate-mediated one-carbon metabolism in solid pattern adenocarcinomas, factors that our analysis suggests are commensurate with poor clinical outcomes. Previous investigations have highlighted the role of extracellular matrix (ECM) alterations, specifically the synthesis of collagen and fibrosis progression in cancer-associated fibroblasts, along with a diminished capacity for collagen degradation, in tumor development and progression (33, 34). Such ECM remodeling can act as a mechanical barrier, constraining the infiltration of immune effector cells into the tumor parenchyma (35). Importantly, our findings posit that tumor cells in solid pattern adenocarcinoma exhibit a predisposition towards epithelial-mesenchymal transition, favoring a more migratory mesenchymal phenotype. This transition is likely contributory to the enhanced metastatic potential observed in these tumors, potentially explaining the elevated incidence rates of pleural invasion, vascular and lymphatic vessel metastasis, as well as lymph node involvement.

The immunological milieu within tumors is pivotal for both the onset and progression of neoplastic disease. Existing literature substantiates that elevated levels of immune infiltration, particularly by CD8^+^T cells and natural killer cells, in the peritumoral region are positively correlated with improved prognosis and therapeutic response (28). Our data corroborate these findings, demonstrating a robust immune response in the tumor microenvironment of solid pattern adenocarcinoma, characterized by pronounced levels of immune cell and antigen-presenting cell infiltration and activity. Nevertheless, in solid pattern adenocarcinoma, an increased expression of immune inhibitory markers, such as PDCD1, PD274, and LAG3, was observed. Concomitantly, there was a substantial influx of immunologically exhausted CD8^+^T cells and regulatory T cells (Treg cells) within the tumor microenvironment (47). These particular immune cells facilitate immune evasion mechanisms. Moreover, the tumor microenvironment in solid pattern adenocarcinoma exhibits a proclivity for negative immune regulation and tolerance, as opposed to other adenocarcinoma patterns. Collectively, these observations point towards an immunosuppressive environment in solid pattern adenocarcinomas, which likely facilitates immune evasion and thereby contributes to distant metastasis. In light of these molecular biological insights, limited surgical resection appears to be an inadequate strategy for managing highly invasive and metastatic solid pattern adenocarcinomas, increasing the risk of disease recurrence. Consequently, a more extensive surgical margin is warranted for these cases. Caution is particularly advised when small-sized tumors display solid pattern adenocarcinoma features. Future research should focus on comparing the efficacy of limited resection and standard lobectomy in terms of overall survival and disease recurrence for this specific adenocarcinoma subtype.

Our investigation is encumbered by several limitations. Primarily, it is a retrospective, single-center study that relies on publicly available transcriptomic datasets, and the cohort of Stage I patients is numerically insufficient, thereby limiting statistical power. Secondly, the clinical data, which were collected within the past five years, lack an extended follow-up period. Future work would benefit from multicenter studies with larger sample sizes and prolonged follow-up to elucidate the prognostic differences between lung segmental resection and pulmonary lobectomy in cases of solid adenocarcinoma. Lastly, this study did not consider another high-grade LUAD subtype, namely micropapillary adenocarcinoma, which also influences surgical decision-making. Its radiographic characteristics warrant investigation in subsequent research.

## 5 Conclusion

In summary, solid pattern adenocarcinoma is characterized by pronounced invasive and metastatic propensities, coupled with notable immune tolerance and evasion mechanisms. These attributes collectively contribute to an unfavorable prognosis and elevated rates of disease recurrence, thereby differentiating this subtype from other forms of lung adenocarcinoma (LUAD). Given these distinct features, the selection of an appropriate surgical approach for solid pattern adenocarcinoma necessitates separate evaluation from other LUAD subtypes.

## Data availability statement

The public data presented in the study are deposited in the TCGA database (https://portal.gdc.cancer.gov/projects/TCGA-LUAD) and OncoSG database (https://src.gisapps.org/OncoSG_public/study/summary?id=GIS031).

## Ethics statement

The studies involving humans were approved by Medical Ethics Committees of Shandong Provincial Hospital. The studies were conducted in accordance with the local legislation and institutional requirements. The participants provided their written informed consent to participate in this study.

## Author contributions

XL: Conceptualization, Data curation, Investigation, Methodology, Writing – original draft, Writing – review & editing. ZG: Conceptualization, Data curation, Investigation, Methodology, Writing – original draft, Writing – review & editing. HD: Data curation, Investigation, Writing – review & editing. CG: Data curation, Writing – review & editing. YY: Data curation, Writing – review & editing. SL: Data curation, Writing – review & editing. ZF: Project administration, Writing – review & editing, Data curation. ZP: Project administration, Writing – review & editing, Conceptualization, Funding acquisition.
