# Supplementary material for: Lung adenocarcinoma: selection of surgical approaches in solid adenocarcinoma from the viewpoint of clinicopathologic features and tumor microenvironmental heterogeneity
Source: Front Oncol. 2024 Mar 5;14:1326626. doi: 10.3389/fonc.2024.1326626 (PMC10949368; doi:10.3389/fonc.2024.1326626)

**Supplementary Material**

**Supplementary Tables**

**Table S1. Validation-LUAD Cohort inclusion and exclusion criteria**

| **Validation-LUAD Cohort Clinical Data** | **Exclude** | **Retention** |
| --- | --- | --- |
| Total cases | — | 3182 |
| Non-primary and non-first diagnosed samples were excluded | 59 | 3123 |
| Neoadjuvant therapy was excluded | 45 | 3078 |
| Micropapillary was excluded | 346 | 2732 |

**Table S2. TCGA-LUAD Cohort inclusion and exclusion criteria**

| **TCGA-LUAD Clinical Data** | **Exclude** | **Retention** |
| --- | --- | --- |
| Total cases | — | 582 |
| Normal tissue was excluded | 58 | 524 |
| Non-primary and non-first diagnosed samples were excluded | 4 | 520 |
| Missing follow-up data were excluded | 9 | 511 |
| Uncertain pathological subtypes | 224 | 287 |
| Micropapillary was excluded | 26 | 261 |
| Samples lacking RNA-seq were excluded | 5 | 256 |

**Table S3. OncoSG-LUAD Cohort inclusion and exclusion criteria**

| **OncoSG-LUAD Clinical Data** | **Exclude** | **Retention** |
| --- | --- | --- |
| Total cases | — | 213 |
| Non-primary and non-first diagnosed samples were excluded | 2 | 211 |
| Uncertain pathological subtypes | 16 | 195 |
| Micropapillary was excluded | 7 | 188 |
| Samples lacking RNA-seq were excluded | 34 | 154 |

**Table S4. TCGA-LUAD demographic and clinicopathological characteristics of patients**

| **Characteristics** | **Solid (N=62)** | **Other (N=199)** | **Total(N=261)** | ***P* value** |
| --- | --- | --- | --- | --- |
| **Age** |  |  |  | 0.02 |
| <60 | 21(36.84%) | 39(20.86%) | 60(24.59%) |  |
| ≥60 | 36(63.16%) | 148(79.14%) | 184(75.41%) |  |
| **Gender** |  |  |  | 0.66 |
| Female | 31(50.00%) | 108(54.27%) | 139(53.26%) |  |
| Male | 31(50.00%) | 91(45.73%) | 122(46.74%) |  |
| **Smoking** |  |  |  | 0.05 |
| Never | 12(21.82%) | 70(37.04%) | 82(33.61%) |  |
| Current/Ever | 43(78.18%) | 119(62.96%) | 162(66.39%) |  |
| **Pathologic_T** |  |  |  | 0.38 |
| T1 | 13(23.64%) | 60(31.91%) | 73(30.04%) |  |
| T2 | 38(69.09%) | 106(56.38%) | 144(59.26%) |  |
| T3 | 2(3.64%) | 14(7.45%) | 16(6.58%) |  |
| T4 | 2(3.64%) | 8(4.26%) | 10(4.12%) |  |
| **Pathologic_N** |  |  |  | 0.07 |
| N0 | 29(53.70%) | 125(68.68%) | 154(65.25%) |  |
| N1 | 14(25.93%) | 32(17.58%) | 46(19.49%) |  |
| N2 | 10(18.52%) | 25(13.74%) | 35(14.83%) |  |
| N3 | 1(1.85%) | 0(0.00%) | 1(0.42%) |  |
| **Pathologic_M** |  |  |  | 1 |
| M0 | 52(94.55%) | 147(94.84%) | 199(94.76%) |  |
| M1 | 3(5.45%) | 8(5.16%) | 11(5.24%) |  |
| **EGFR driver mutations** |  |  |  | 0.4 |
| No | 17(80.95%) | 69(69.00%) | 86(71.07%) |  |
| Yes | 4(19.05%) | 31(31.00%) | 35(28.93%) |  |
| **Pathologic_stage** | |  |  | 0.69 |
| Stage I | 32(53.33%) | 108(55.38%) | 140(54.90%) |  |
| Stage IIA-IIIA | 22(36.67%) | 74(37.95%) | 96(37.65%) |  |
| Stage IIIB-IV | 6(10.00%) | 13(6.67%) | 19(7.45%) |  |

**Table S5. OncoSG-LUAD demographic and clinicopathological characteristics of patients**

| **Characteristics** | **Solid(N=29)** | **Others(N=159)** | **Total(N=188)** | ***P* value** |
| --- | --- | --- | --- | --- |
| **Age（60）** | |  |  | 0.76 |
| <60 | 10(34.48%) | 47(29.56%) | 57(30.32%) | |
| ≥60 | 19(65.52%) | 112(70.44%) | 131(69.68%) | |
| **Gender** |  |  |  | 0.45 |
| Female | 14(48.28%) | 92(57.86%) | 106(56.38%) | |
| Male | 15(51.72%) | 67(42.14%) | 82(43.62%) | |
| **Smoking** |  |  |  | 0.06 |
| No | 14(48.28%) | 109(68.55%) | 123(65.43%) | |
| Yes | 15(51.72%) | 50(31.45%) | 65(34.57%) | |
| **Clinical _ stage** | |  |  | 6.50E-03 |
| I | 11(37.93%) | 102(64.97%) | 113(60.75%) | |
| II | 6(20.69%) | 26(16.56%) | 32(17.20%) | |
| III | 8(27.59%) | 25(15.92%) | 33(17.74%) | |
| IV | 4(13.79%) | 4(2.55%) | 8(4.30%) |  |
| **Histological _ Grade** | | |  | 1.90E-21 |
| Well differentiated | 1(3.85%) | 14(9.93%) | 15(8.98%) |  |
| Well to Moderately differentiated | 0(0.00%) | 4(2.84%) | 4(2.40%) |  |
| Moderately differentiated | 5(19.23%) | 116(82.27%) | 121(72.46%) | |
| Moderately to Poorly differentiated | 1(3.85%) | 5(3.55%) | 6(3.59%) |  |
| Poorly differentiated | 19(73.08%) | 2(1.42%) | 21(12.57%) | |

**Supplementary Figures**

Figure S1. Heterogeneity in cell proliferation, invasive capacity, and metabolism between solid pattern and other patterns in the OncoSG cohort


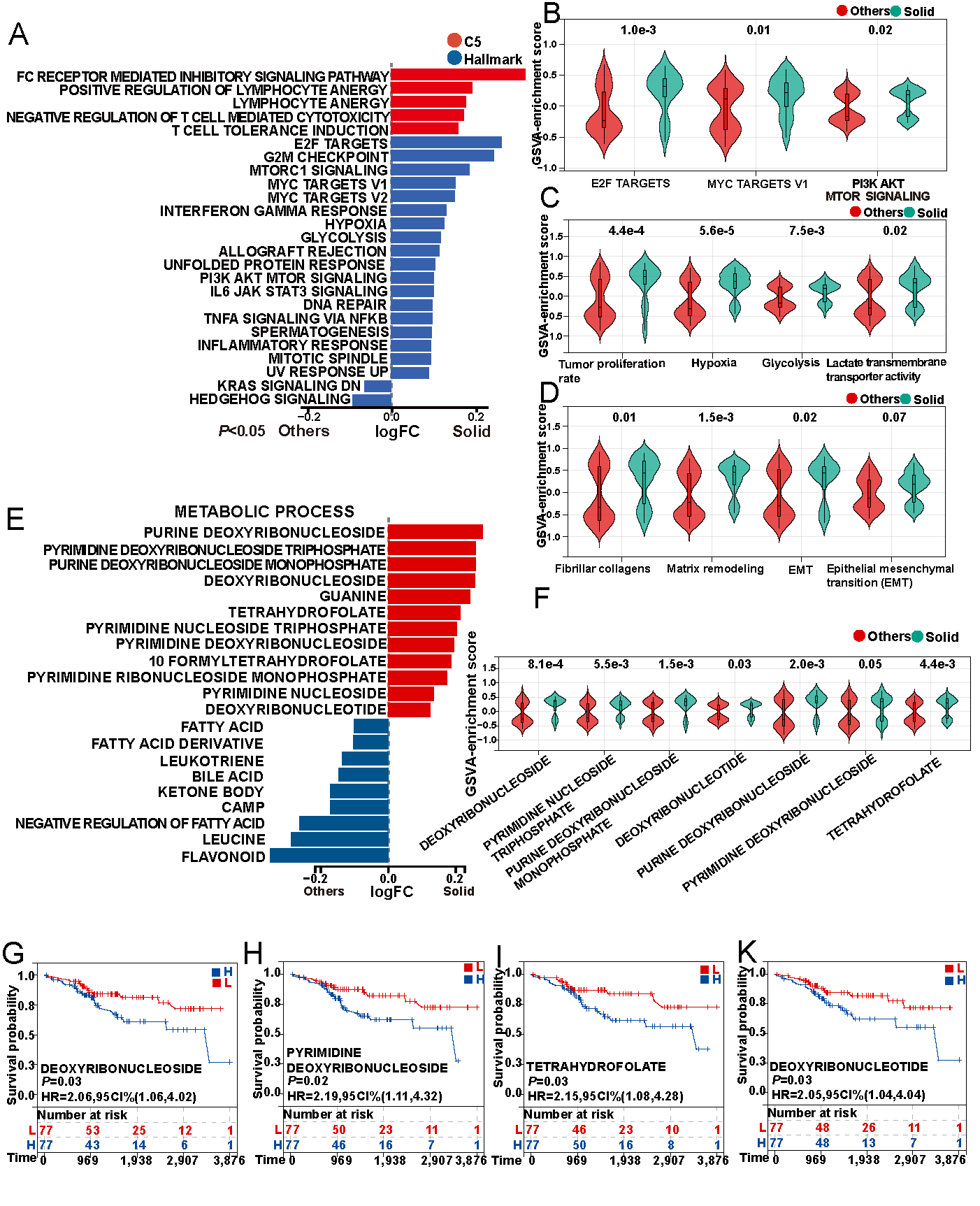


(A) Gene Set Variation Analysis (GSVA) reveals differences in the enrichment scores of Hallmark gene sets and C5 gene sets between the two groups. (B) Violin Plot shows differences in the enrichment scores of biomarkers associated with invasion and metabolism between the two groups. (C) Violin Plot shows the differences in cell proliferation rate, hypoxia, glycolysis and lactate transmembrane transporter activity scores between the two groups. (D) Violin Plot shows the differences in collagen fiber transcription score and extracellular matrix remodeling score between the two groups. (E) Gene Set Variation Analysis (GSVA) reveals significant differences in the enrichment of metabolic process gene sets between the two groups. (F) Violin Plot shows differences in nucleotide and tetrahydrofolate metabolism between the two groups. (G)，(H), (I), and (J) Kaplan-Meier analysis of deoxyribonucleoside, pyrimidine deoxyribonucleoside, tetrahydrofolate, and deoxyribonucleotide.

Figure S2. Survival heterogeneity between the active and inactive groups of nucleotide metabolism in the OncoSG cohort


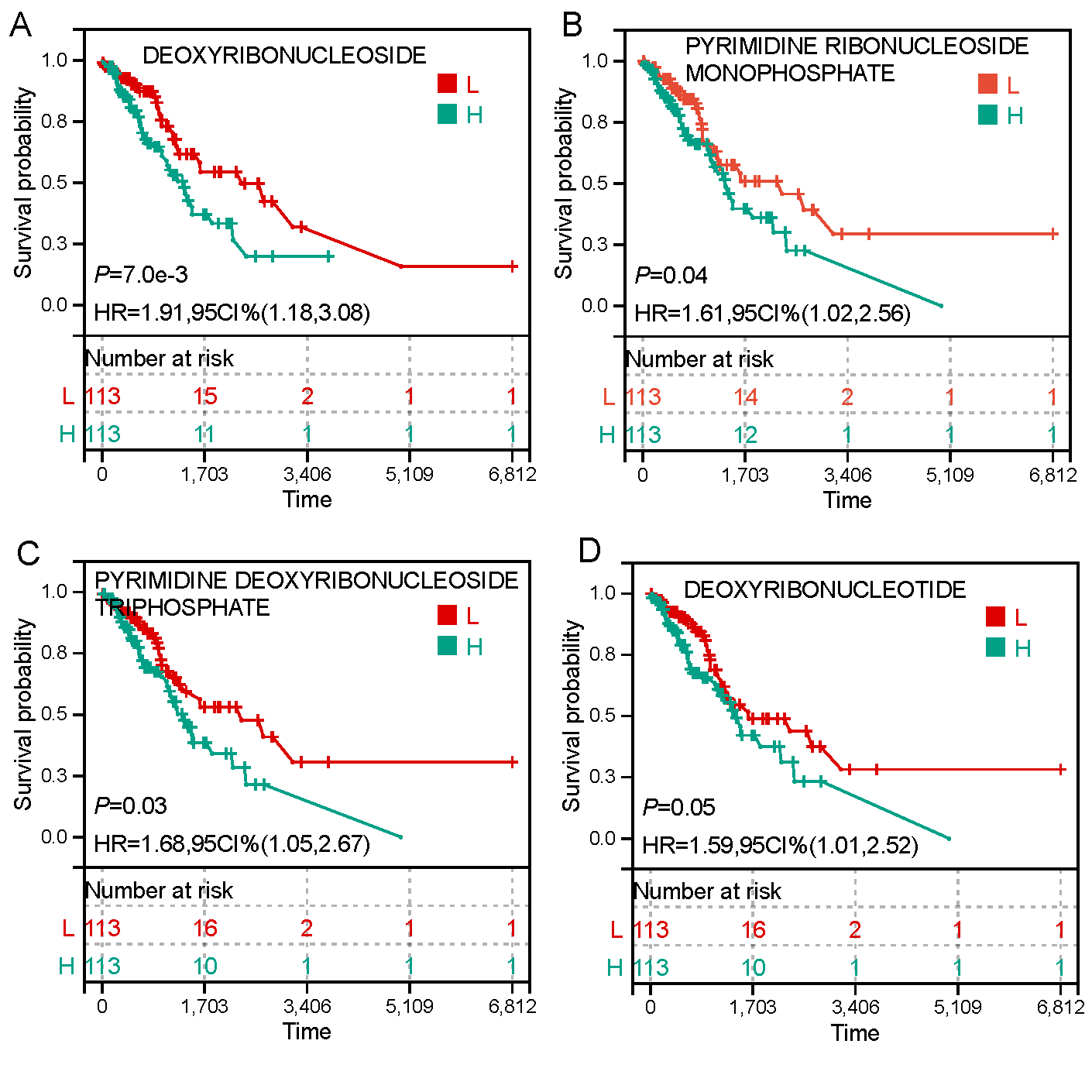


(A), (B), (C), and (D) Kaplan-Meier analysis of deoxyribonucleoside, pyrimidine ribonucleoside monophosphate, pyrimidine deoxyribonucleoside triphosphate, and deoxyribonucleotide.

Figure S3. Immune heterogeneity between solid pattern and other patterns in the OncoSG cohort


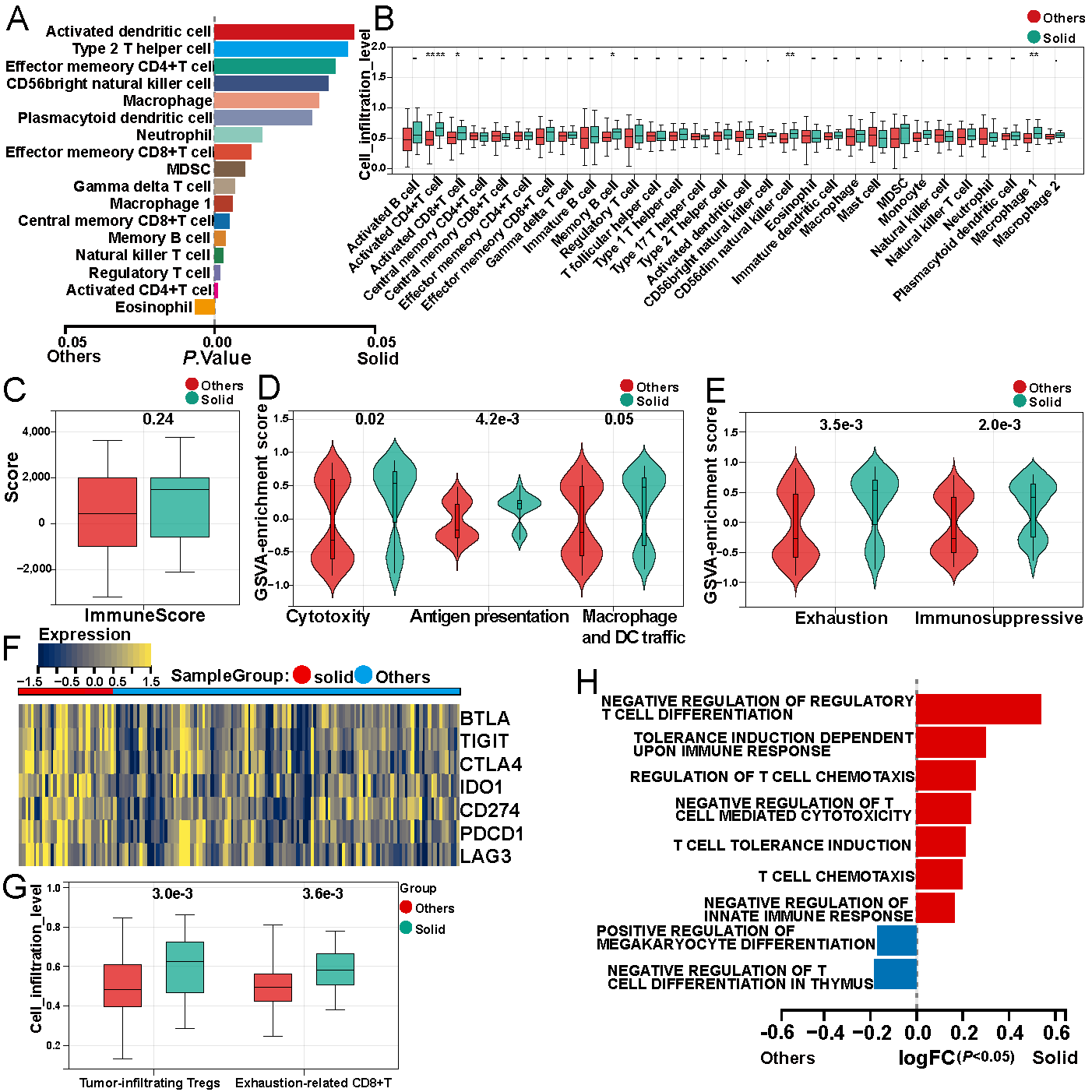


(A) Single-sample Gene Set Enrichment Analysis (ssGSEA) reveals differences in immune cell infiltration scores between the two groups. (B) Box Plot shows differences in immune cell infiltration abundance between the two groups. (C) Violin Plot shows differences in immune scores between the two groups. (D) Violin Plot shows differences in cytotoxicity scores between the two groups. (E) Violin Plot shows differences in tumor microenvironment immune exhaustion scores between the two groups. (F) Heatmap of differentially expressed immune checkpoints between the two groups. (G) Box Plot shows differences in quantification of tumor-infiltrating regulatory T cells (Tregs) and exhausted CD8^+^T cell within the tumor microenvironment between the two groups. (H) Gene Set Variation Analysis (GSVA) shows significant differences in immune tolerance, immune suppression, and immune cell physiological functions between the two groups.

Figure S4. Multivariate survival analysis in the OncoSG cohort shows that the solid pattern pathological subtype is an independent risk factor for overall survival


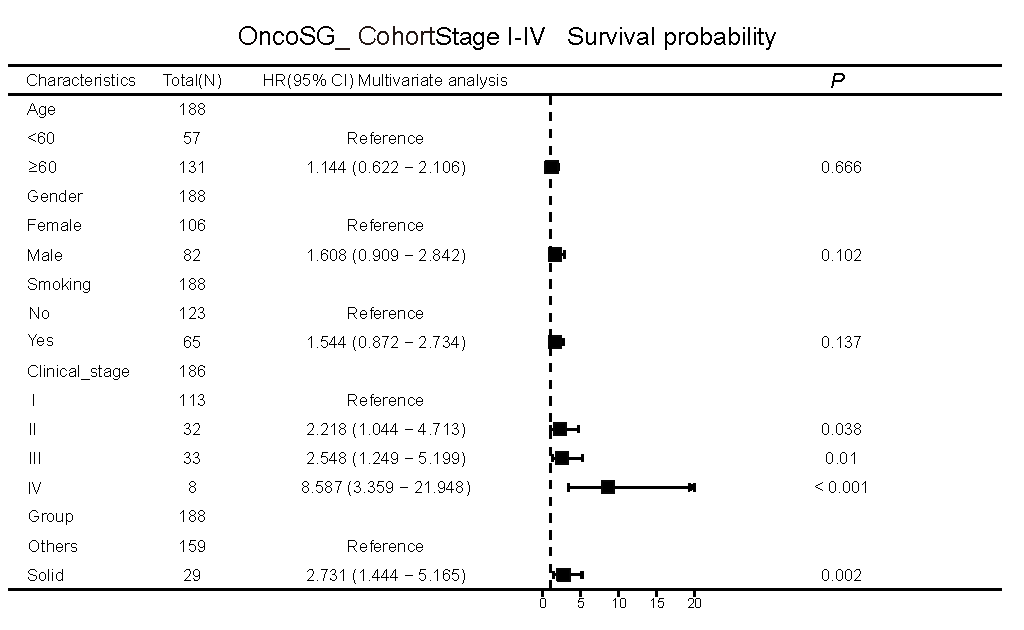

Supplement: Supplementary file 1 [file DataSheet_1.docx]
